# Evolution and influencing factors of China's rural population distribution patterns since 1990

**DOI:** 10.1371/journal.pone.0233637

**Published:** 2020-05-29

**Authors:** Hua Zhang, Simeng Zhang, Zheda Liu

**Affiliations:** Beijing Key Laboratory of Environmental Remote Sensing and Digital Cities, Faculty of Geographical Science, Beijing Normal University, Beijing, China; Institute for Advanced Sustainability Studies, GERMANY

## Abstract

Population is a major production factor in rural development in China, which makes the study of rural population distribution patterns at different times and the factors influencing the population distribution an important foundation for understanding the issues in rural China and moving forward with the implementation of rural revitalization strategies. This paper analyzed the spatial evolution of the population in rural China based on population census data for the People’s Republic of China by county in 1990, 2000 and 2010. Applying the geographical detector method, this paper also delved into the contributing factors that influenced the distribution based on the natural, social and economic data, such as the potential crop productivity, the average slope, the urbanization rate and the time cost to reach the nearest cities. The results indicate that the migration of the population from the rural areas into the cities, which was a result of rapid urbanization, did not change the original population distribution in rural China significantly. The rural population was still concentrated in the eastern plains, basins and deltas, and the North China Plain and Sichuan Basin still house the bulk of rural residents, but the population density of rural residents in the North China Plain and Sichuan Basin decreased from 1990 to 2010. The rural population in China tended to be distributed around the cities. Seventy-four percent of the rural population lived in an area within a 60-minute driving distance from the surrounding cities. The areas with dense rural population were basically consistent with the locations of the current major urban agglomerations in China. The current distribution of the rural population in China was a result of natural, social and economic conditions and location factors. Among them, natural factors such as the potential crop productivity and the degree of surface fragmentation had the most significant influence.

## Introduction

The development of urbanization in China has accelerated since the implementation of the reform and opening-up. The urbanization rate has increased from 17.92% in 1978 to 59.58% in 2018 [1]. Along with the rapid urbanization process, large-scale rural-urban migration leads to the rapid decline in rural population [2–5]. The rural population in China decreased from 790 million in 1978 to 564 million in 2018 [1]. However, other geographical elements of rural areas, such as land, cannot quickly adapt to this change in rural population due to policies, institution and other reasons [6, 7]. The dislocation of demographic and other geographical elements has produced a series of rural problems, such as hollowing villages [8–10], farmland abandonment [11–14], and rural decline [2, 15]. The mass rural-urban migration and rapid urban growth of China have also caused the unprecedented loss of arable land [16]. Under the strategic background of rural revitalization, rural areas has been endowed with new development opportunities, which means that rural resource allocation should correspond to the distribution pattern of rural population. The rational distribution of infrastructure, public services and the development of rural industries is inseparable from the trends in the rural population distribution. Therefore, based on the reality of the large-scale reduction in rural population, analysis of the changes in the distribution patterns of the rural population and the influencing factors helps to improve the understanding of the trends in rural population distribution, which are significant for the implementation of the rural revitalization strategy and sustainability in rural areas.

China's population distribution patterns and the evolution of these patterns are the focus of research on population geography [17, 18]. A large number of existing studies have revealed that China's population distribution patterns are formed by the combination of natural environmental and socioeconomic conditions. Natural environmental factors mainly include topography, climate, and water resources [19–21]; socioeconomic conditions include economic development level, industrial structure, and accessibility [20]. However, few studies have attempted to uncover changes in the spatial patterns of rural population and their influencing factors in China. At present, research on rural population issues has predominantly concentrated on two perspectives. On the one hand, the research has mainly focused on the changes in rural population and its driving factors [22], the changes in rural labor [23–26], the decline in the rural population caused by rural-urban migration and the reasons for this migration [3, 27, 28]. These studies often use typical rural regions as cases to argue that some rural areas experience dramatic rural population decline while others experience growth [22]. On the other hand, some studies have noted the negative effects of rural population decline in emigration regions. The research has mainly investigated the effects that have appeared after the changes in the rural population, such as hollowing villages [10], split-household arrangements [29] and aging populations [30, 31].

The insufficiency of these studies is that they do not pay enough attention to the spatial issues of rural population changes, including the spatial distribution patterns, changes from dense to sparse populations, and so on. With the process of rapid urbanization, a large number of people migrate from rural to urban area. Whether the distribution patterns of rural population has been changed during the urbanization process is of great significance. Meanwhile, agriculture is the main industry in rural areas, where the spatial distribution patterns of the populations should have special influential factors, such as the distance from the city and the natural agricultural conditions. Existing research on the factors affecting the spatial distributions of the population can be referenced for the exploration of the factors influencing the spatial distribution pattern of rural population, but it is still necessary to explore those factors, specifically in rural areas.

This paper has two aims. First, this paper explores the spatial evolution of China's rural population to determine whether the rural-urban migration brought by rapid urbanization has changed the original spatial patterns of the rural population in China. Second, based on the relevant population distributions theories and the particularities of rural areas, this paper uses natural, socioeconomic and location indicators with the geographical detector method to explore the key factors that shape the spatial patterns of the rural population in China. This paper may improve the understanding of the process of the changes in the spatial distributions of rural population in developing countries and provide a reference for the implementation of rural revitalization strategies and sustainable development in rural areas.

## Data and methods

### Data

The rural population refers to the permanent resident population in the countryside, excluding the population living in the city and town. So the rural population is relative to the urban population. In this paper, the county-level rural population data comes from the population census of the People’s Republic of China in 1990, 2000 and 2010. Specifically, the number of a county’s rural population corresponds to the “rural population” field in the census data.

Basic geographic statistics, such as the vector boundaries of the county-level administrative units, digital elevation model (DEM) and potential crop productivity, are all sourced from the Resource and Environmental Science Data Center of the Chinese Academy of Sciences (http://www.resdc.cn). Among them, the vector boundaries of the county-level administrative units in 1990 and 2000, which are involved in the administrative division adjustment, are merged and mapped based on the boundaries in 2010. The socioeconomic data, such as the GDP of the primary industry in the county, the regional industrial production value, and the average net income of the rural residents, come from the “China statistical yearbook for region economy 2011”.

### Research methods

This paper utilized the population agglomeration index to measure the degree of rural population concentration [32] and to compare the concentration of rural population within a region to that of the country. It can be calculated with the following formula (1):

$${JJD}_{i}=\frac{(P_{i}/P)*100\%}{(A_{i}/A)*100\%}=\frac{P_{i}/A_{i}}{P/A}$$ (1)

where JJD_*i*_ represents the rural population concentration in the *i* region, P_*i*_ represents the number of rural population in the *i* region (person), A_*i*_ represents the land area in the *i* region (km^2^), A is the total land area of all the regions (km^2^), and P is the total rural population of all the regions (person).

This paper uses the geographical detector method to analyze the influencing factors of the distribution patterns of the rural population. Spatial heterogeneity is one of the basic characteristics of geography [33]. Geographical detectors are a new set of statistical methods that detect the degree of stratified spatial heterogeneity and reveal its driving factors from the perspective of spatial heterogeneity [34]. The core idea is that if an independent variable is an important factor affecting the dependent variable, it should have a similar spatial distribution to the dependent variable. The method has been used to measure the stratified spatial heterogeneity of existing data and to explore the influencing factors. Compared with the traditional mathematical statistics and spatial analysis methods, this method has no linear assumptions on the variables, and its principles can guarantee immunity to multi-collinearity. On the other hand, if the independent variables and the dependent variables are both numerical, after the discretization processing of the independent variable to convert it into a categorical variable, the relationship between the dependent variable and the independent variable constructed by the geographical detector method is more reliable than a classic econometric regression [35]. Based on these assumptions, using the geographical detector method, this paper takes the county as the basic analysis unit to explore the main factors affecting the spatial distribution patterns of rural population. The model is shown in formula (2) as follows:

$$q_{D,P}=1-\frac{1}{n\sigma_{P}^{2}}\sum_{i=1}^{m}n_{D,i}\sigma_{P_{D,i}}^{2}$$ (2)

where *q_D,P_* is the detection force index of the influencing factor D for the rural population distribution patterns; *n_D,i_* and *n* are the number of units in *i* sub-region and the number of units in the whole region, respectively; *m* is the number of sub-regions; $\sigma_{P}^{2}$ is the variance of the rural population density in the whole region; and $\sigma_{P_{D,i}}^{2}$ is the variance of the rural population density in the *i* sub-region. The value interval of *q_D,P_* is [0, 1]. Larger values indicate more obvious stratified spatial heterogeneity of the rural population density. If the stratification is generated by the influencing factor D of the rural population density, the larger *q_D,P_* indicates that the influencing factor has stronger explanatory power for the spatial differentiation in the rural population density, and vice versa. If the value of *q_D,P_* is 1, the influencing factor D completely controls the spatial distribution of the rural population. If the value of *q_D,P_* is 0, the influencing factor D has no relationship with the spatial distribution of the rural population. The *q_D,P_* value indicates that the influencing factor D explains the spatial distribution characteristics of the rural population of 100 × q%.

## Evolution of rural population distribution pattern

### The agglomeration pattern of rural population was obvious and had changed little

**Fig 1:**
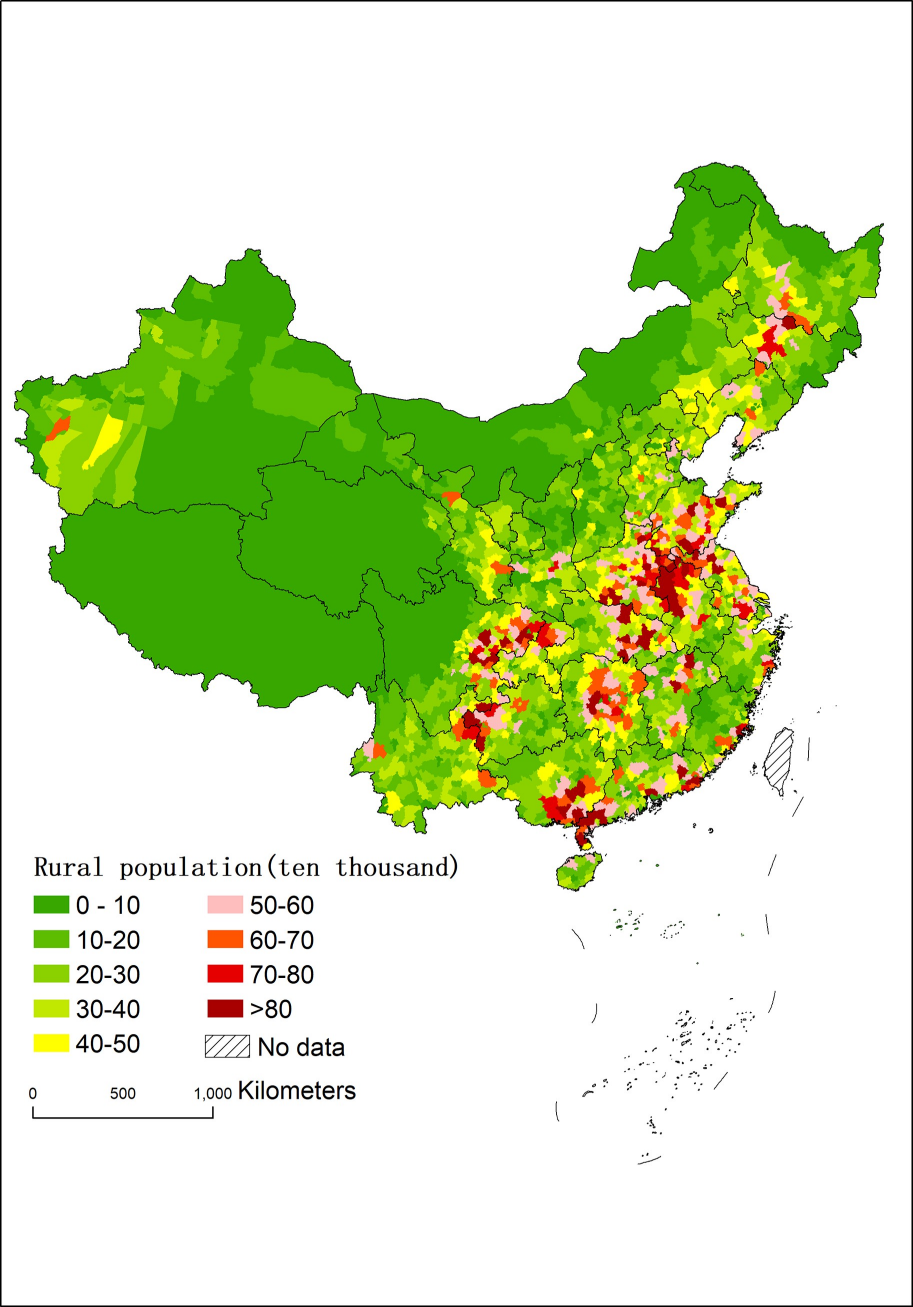
Rural population size of China in 2010 at the county level.

In terms of the rural population size, the agglomeration of rural population was obvious and the rural population was mainly distributed in the plains, basins and deltas with superior terrain condition such as the Northeast Plain, the North China Plain, the middle and lower reaches of the Yangtze River, the Sichuan Basin, the basins in the southwestern Sichuan and the middle of Yunnan, and the Pearl River Delta (Fig 1). In 2010, the top five counties with the largest size of rural population were Linquan County in Anhui Province, Puning city in Guangdong Province, Zhenxiong County in Yunnan Province, Huoqiu County in Anhui Province, and Lianjiang city in Guangdong Province. All of them, which had rural population of more than 1 million, were located on terrains with superior conditions. While the top counties with smallest size of rural population were mainly in Inner Mongolia, Tibet and Qinghai Province. The smallest size of rural population were in the Aksay Kazakh Autonomous County in Gansu, the Huolin Gol city in Inner Mongolia and Manzhouli in Inner Mongolia, where the rural population were below 1,500.

**Fig 2:**
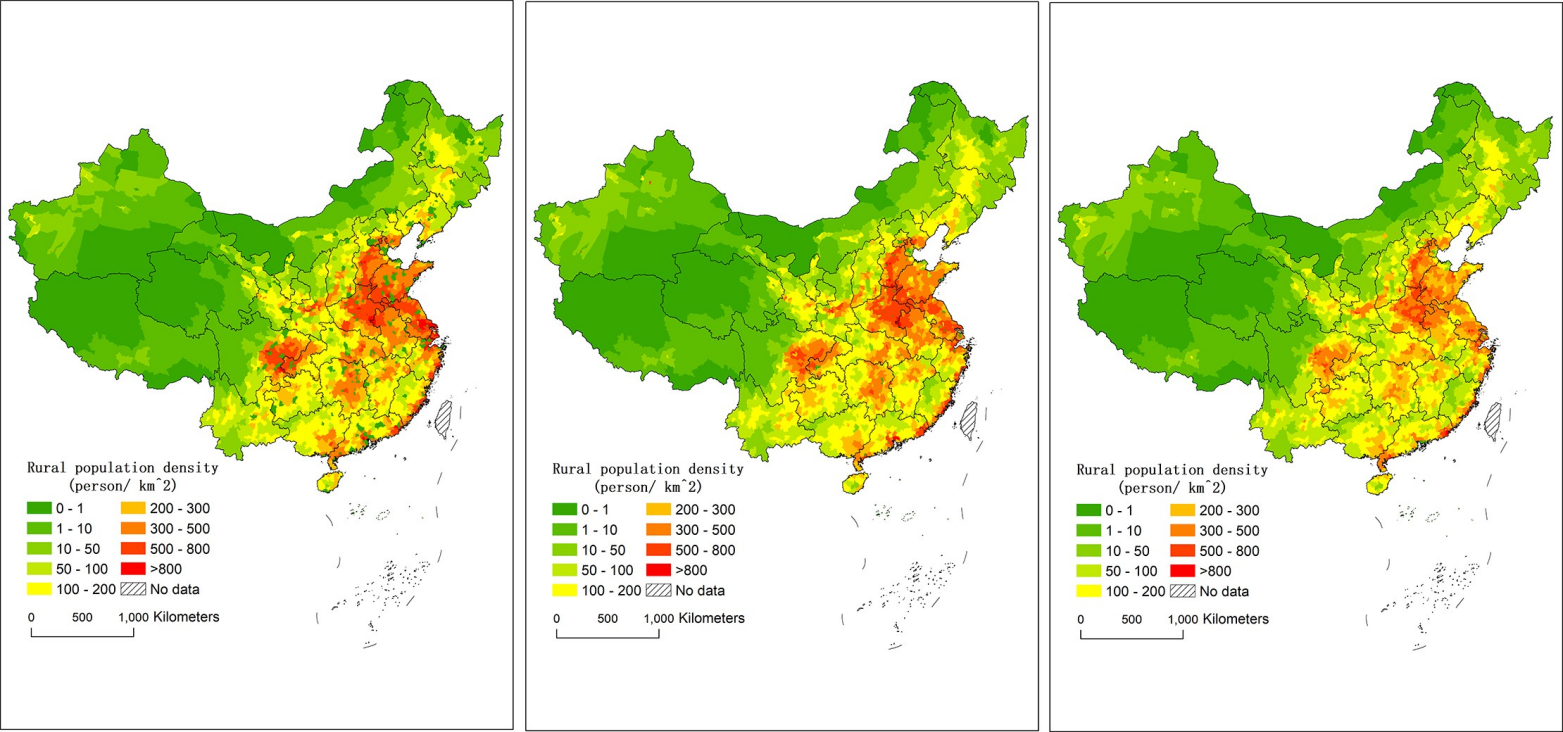
Rural population density of China from 1990 to 2010 at the county level. a. in 1990, b. in 2000, c. in 2010.

In terms of the rural population density, the areas with highest rural population density were mainly distributed in the plains, basins and deltas in Southeast China that have superior terrain conditions (Fig 2). The North China Plain, the middle and lower reaches of the Yangtze River and the Sichuan Basin were the rural population centers. The population density of the low-lying or near-water areas, such as the North China Plain, the middle and lower reaches of the Yangtze River, the Sichuan Basin, the Yangtze River Delta, and the Pearl River Delta in the eastern region, had more than 200 persons/km^2^, which is a high density for rural population. The population density of the rural areas in the northwestern region is less than 100 persons/km^2^, which is sparse for rural population.

From 1990 to 2010, although the size of rural population continued to decrease, the spatial distribution patterns of the rural population size and rural population density did not change significantly. In terms of the total size, the rural population in China in 1990, 2000 and 2010 were 830 million, 780 million and 660 million, accounting for 73.7%, 63.1% and 49.7% of the total population, respectively. This paper calculated the mean center and standard deviational ellipse of the China’s rural population distribution by the ARCGIS software. The mean center of the rural population has always been between 110.93°E and 113.06°E or between 32.36°N and 33.38°N, with little variation. Furthermore, based on the standard deviational ellipse of the total population on the county level in the rural areas, the results showed that the range, long axis, short axis and the rotation angles of the standard deviational ellipse coverage in 1990, 2000 and 2010 were not obviously changed (Table 1). The stability of the mean center and standard deviational ellipse illustrates that the spatial distribution pattern of the total rural population was basically stable. In terms of rural population density (Fig 2), the counties with population densities of more than 300 persons/km^2^ were mainly concentrated in the North China Plain and the Sichuan Basin in 1990, 2000 and 2010, which were the two centers of the rural population.

**Table 1:** Main characteristic values of population standard deviational ellipse of rural population.

	Long axis (km)	Short axis (km)	Rotation angle
1990	1035.45	767.22	34.06°
2000	1044.72	794.71	34.84°
2010	1078.12	835.26	35.92°

### The concentration degree of rural population was decreasing and lower than that of the total population

At the national level, the rural population tended to be concentrated in some counties, but the degree of concentration has declined. First, the counties with population densities of 300 to 500 persons/km^2^ had 550 million, 480 million, and 360 million people in 1990, 2000 and 2010, accounting for 32.92%, 30.81% and 27.38% of the rural population and separately containing 927, 926, and 842 county-level administrative units, respectively. In counties with a population density greater than or equal to 500 persons/km^2^, the rural population in 1990, 2000, and 2010 were 430 million, 360 million, and 220 million, accounting for 26.07%, 22.89% and 16.92% of the rural population and containing 683, 640 and 494 county-level administrative units, respectively. Then, according to the rankings of all counties based on the volumes of the rural population sorted from the top to the bottom, in 1990, 2000, and 2010, the top 30% of the counties contained 62.35%, 59.81%, and 58.2% of the rural population, respectively; that is, the rural population was concentrated at the national level. On the other hand, the proportion of the rural population that was concentrated in the top counties has declined. First, from 1990 to 2010, the proportion of the rural population in the top 10% of the counties decreased by approximately 1.4% every 10 years (Table 2). The proportion in the top 20% of the counties fell by approximately 2.3% in the first 10 years and decreased by 1.5% in the last ten years. The proportion of the rural population in the top 30% of the counties fell by approximately 2.5% in the first decade and by approximately 1.6% in the next decade.

**Table 2:** Statistics of rural population distribution in China during 1990–2010.

Rank of counties	1990		2000		2010	
	Proportion of rural population	Proportion of total population	Proportion of rural population	Proportion of total population	Proportion of rural population	Proportion of total population
Top 100	14.46%	15.92%	13.75%	18.17%	13.28%	21.33%
Top 238(10%)	29.05%	29.10%	27.59%	31.21%	26.24%	33.82%
Top 475(20%)	48.04%	46.46%	45.79%	47.91%	44.33%	49.97%
Top 713(30%)	62.35%	59.69%	59.81%	60.79%	58.20%	62.50%

The rank of the counties is based on the size of the rural population or total population sorted from the top to the bottom.

Since 2000, the agglomeration of the rural population has been lower than that of the total population, and the trend has been increasing. The ranking of the countries across China based on the rural population and total populations in 1990, 2000, and 2010 showed that for the top-ranked counties in 1990, the differences between the ratio of the rural population and those of the total population were minimal, and the former was higher than the latter even for the top 20% and 30% of the counties (Table 2). However, the concentration of the rural population decreased, while the concentration of the total population increased; this phenomenon varied significantly in 2000. In 2000, the proportion of the rural population in the top 100, 10%, 20% and 30% of counties was lower than that of the total population by the ratios of 4.42%, 3.62%, 2.12%, and 0.98%, respectively, and the gap in 2010 increased by 8.05%, 7.58%, 5.64%, and 4.30%, respectively.

### The rural population tends to be distributed around cities

Following the national population classification criteria from Liu Ruiwen [32], this paper uses formula (1) to calculate the rural population agglomeration index at the county level. The classification criteria for rural population agglomeration index are shown in Table 3.

**Table 3:** The classification standard of China's rural population agglomeration index.

Classification of rural areas		Rural population aggregation index
Densely populated rural region	Highly dense area	JJD≥ 8
	Moderately dense area	4 ≤ JJD < 8
	Lowly dense area	2 ≤ JJD < 4
Mean populated rural region	Area above the national average	1 ≤ JJD < 2
	Area slightly below the national average	0.5 ≤ JJD < 1
Sparsely populated rural region	Relatively sparse area	0.2 ≤ JJD < 0.5
	Absolutely sparse area	0.05 ≤ JJD < 0.2
	Extremely sparse area	JJD < 0.05

**Fig 3:**
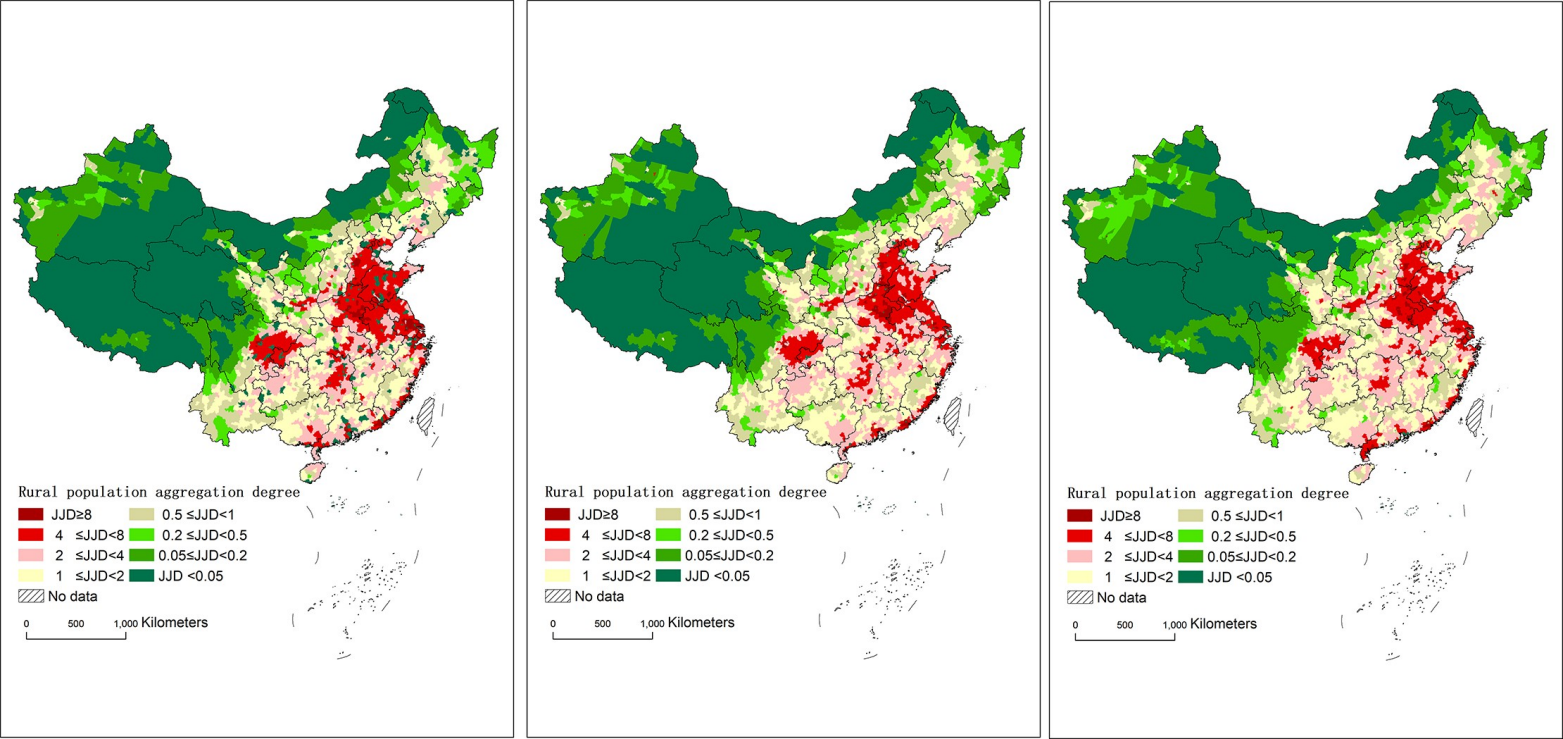
Rural population aggregation index (JJD) at the county scale in China during 1990–2010. a. in 1990, b. in 2000, c. in 2010.

According to the spatial distribution map of the rural population agglomeration (Fig 3), the dense areas of rural population have basically aligned with the current major urban agglomerations in China since 1990 and present multi-circle agglomeration characteristics supported by the cities. In the southeastern half of Hu’s line, there is a ringed structure in the rural population that contains a high-density area, a moderate-density area, and a low-density area–area above the national average. If taking the high-density area and the moderate-density area as the core of the circle, it is basically consistent with the current major city clusters in China. The moderate-density areas for the rural population (4 ≤ JJD < 8) and the high-density areas (JJD ≥ 8) were mainly located in the city clusters of Beijing-Tianjin-Hebei, Zhongyuan, Shandong Peninsula, Guanzhong, the Yangtze River Delta, Chengdu-Chongqing, etc. The low-density areas (2 ≤ JJD < 4) mainly included the city clusters of Harbin-Changchun, central and southern Liaoning, the middle reaches of the Yangtze River, the Pearl River Delta, and the Beibu Gulf. In the northwest half of Hu’s line, the spatial distribution of the rural population showed an irregular circle structure: the area was slightly below the national average and contained a relatively sparse area, an absolutely sparse area, and an extremely sparse area. The rural population was concentrated in the areas with superior natural conditions, such as basin oases and the valley plains between mountains.

**Fig 4:**
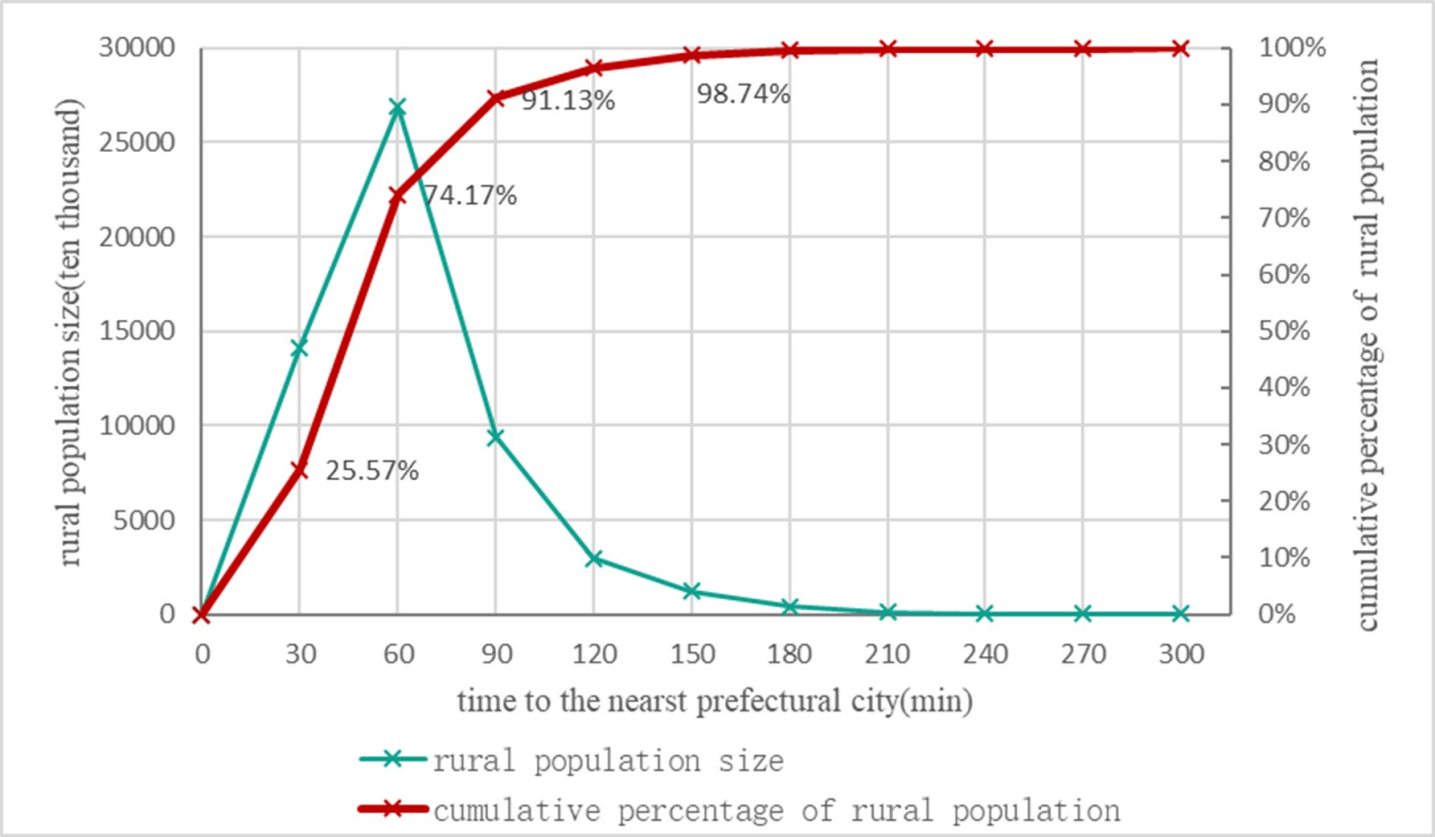
The relationship between the number of rural population and distance to the nearest prefecture-level districts in China in 2010.

Based on the discovery of the relationship between the distribution characteristics of the rural population and the city clusters, the curve of the relationship between the urban distance and the number of rural people is shown in Fig 4, which indicates that there was significant clustering of the rural population close to the urban areas. Specifically, this paper first used the 2013 national road network data, including the high-speed roads, national roads, provincial roads, county roads, and township roads, which were assigned speeds of 100 km/h, 80 km/h, 60 km/h, 50 km/h, and 30 km/h, respectively. Areas without any roads were assigned a 10 km/h walking speed. Then, based on the lowest cumulative cost distance from each cell to the nearest prefecture-level city, which was calculated in ArcGIS, the final average value calculated in the county represented the distance from each county to the nearest prefecture-level city. Finally, with the time to the nearest prefecture-level city as the horizontal axis and the cumulative percentages of the rural population as the vertical axis, the statistical results show that the 25.57%, 74.17%, 91.13%, 96.51% and 98.74% of the rural population was concentrated in the regions that were 30 minutes, 60 minutes, 90 minutes, 120 minutes and 150 minutes to the prefecture-level city. It can be said that the concentration of the rural population near the cities is an important basis for the development of urban and rural integration. Rural development can rely on cities, and cities may also be important fulcrums for rural revitalization.

## Influential factors of the rural population spatial patterns

### Factor selection

Two group of factors affecting the spatial patterns of the rural population are considered in this paper, one is the general factors affecting the population distribution and the other is the special factors of the rural areas. Existing studies have indicated that the former mainly includes societal, historical, natural environmental and economic conditions [2], while the spatial differences in the changes of the rural population are mainly related to economic factors, including the per capita GDP, urbanization level, etc. Noneconomic factors, such as the rural population size, also affect the population distribution patterns [22]. The latter considers that the people in rural areas are mainly engaged in agricultural production. The distribution of the rural population is closely related to the agricultural production conditions. Therefore, the natural conditions and the location relationship with the city may have more significant impacts.

Therefore, from the three dimensions included natural environmental factors, socioeconomic factors and location conditions, this paper selected 13 indicators and constructed a quantitative model to explore the factors influencing the spatial patterns of rural population (Table 4). The natural environmental factors mainly reflect the suitability of land for agricultural production and residence from the perspectives of topography and land cultivation quality. Potential productivity for crops was used to measure the land cultivation quality and cropland acreage per capita was used to proxy the amount of arable land. This paper also used average slope and surface fragmentation based on the DEM to proxy the suitability of land for agricultural production. The socioeconomic factors mainly reflect the “pull force” of the city to the rural population through economic development and the “push force” of rural economic status to the rural population. This paper used urbanization rate, gross industrial production and GDP per capita to proxy the pull force of the city to the rural-urban migrants and gross product of the primary industry and per capita net income of rural residents to proxy the push force of rural area to the rural-urban migrants. The location factors mainly reflect the impact of location on the distribution of the rural population from the perspective of the spatial relationship with the city. The nearest prefecture-level city and provincial capital city is the center of attraction for rural-urban migrants. So the paper used time to the nearest prefecture-level city and time to provincial capital to proxy the location of the county. Considering that the rural population will be affected by natural growth and the number of original rural population, this paper added two indicators: the natural growth rate in 2010 and the number of rural population in 2000.

**Table 4:** The factors for the spatial pattern of rural population.

Classification of factors	Code of factors	Implication
Natural environmental factors	X1	Potential productivity for crops
	X2	Average slope
	X3	Surface fragmentation
	X4	Cropland acreage per capita
Socio-economic factors	X5	Urbanization rate in 2010
	X6	Gross product of the primary industry in 2010
	X7	Gross industrial production in 2010
	X8	GDP per capita in 2010
	X9	Per capita net income of rural residents in 2010
Location factors	X10	Time to the nearest prefecture-level city
	X11	Time to provincial capital
Other factors	X12	Annual natural growth rate in 2010
	X13	Number of rural population in 2000

### The results of the factor detectors

First, based on the China county-level data in 2010, this paper used the factor detectors of geographical detector method and took the rural population density of the county as a dependent variable to detect the explanatory power of each factor on the spatial distribution of the rural population. The method aimed to indicate the importance of the indicators on the spatial distribution patterns of the rural population. The results are shown in Table 5.

**Table 5:** The q-value of factors affecting rural population distribution in China.

	X1	X2	X3	X4	X5	X6	X7	X8	X9	X10	X11	X12	X13
q statistic	0.45	0.32	0.37	0.22	0.05	0.23	0.25	0.04	0.12	0.17	0.22	0.03	0.34
p value	0.00	0.00	0.00	0.00	0.00	0.00	0.00	0.00	0.00	0.00	0.00	0.00	0.00

The factor detectors detected the extent to which each factor X explained the spatial stratified heterogeneity of the rural population by calculating the q value. The q values were sorted from large to small as follows: the potential crop productivity (0.45)>surface fragmentation (0.37)>number of rural population in 2000 (0.34)>average slope (0.32)>gross industrial production in 2010 (0.25)> gross product of the primary industry in 2010 (0.23)>time to provincial capital (0.22)> cropland acreage per capita (0.22)> time to the nearest prefecture-level city (0.17)> per capita net income of rural residents in 2010 (0.12)> urbanization rate in 2010 (0.05)> GDP per capita in 2010 (0.04)> annual natural growth rate in 2010 (0.03).

The natural environmental factors were the most important factors influencing the formation of the spatial pattern of the rural population. Three of the top five q-values were natural environmental factors, namely, the top two factors were potential crop productivity and the surface fragmentation degree plus average slope ranked fourth. In particular, for the potential crop productivity factor, its q value can reach 0.45, which means that the explanatory power for the spatial pattern of the rural population is 45%. The natural environmental factors were the most significant factors affecting the formation of the spatial patterns of the rural population. These factors not only determine the suitability of the residence but also are the fundamental factors influencing agricultural production. In areas with a high potential agricultural production and a low degree of habitat fragmentation, the stages of the agricultural production capacity and agricultural economic development are advanced, allowing the area to carry a large number of rural population based on the large capacity for rural employment. This leads to the concentration of the rural population.

The original rural population distribution pattern also was a very important influencing factor. The q-value of the rural population in 2000 was ranked third at 0.34. It means that 34% of the spatial pattern of the rural population can be explained by the original rural population distribution. That is, the current spatial pattern of the rural population was still significantly affected by the original pattern. Although the rural-urban migration was one of the important forces in reshaping the spatial patterns of the rural population, due to the large rural population base, path dependence still had an obvious effect on the spatial pattern of rural population. Therefore, the agglomerations of rural population should currently be the key regions for rural revitalization.

The economic and location factors also had significant impacts on the spatial pattern of the rural population, while the natural growth rate had little impact. Among the economic factors, the regional industrial production value and GDP of the primary industry in 2010 were 0.25 and 0.23, respectively. Among the location factors, the q-values of the times to the capital cities of each province and the times to the nearest prefecture-level city were 0.22 and 0.17, respectively, which again showed the relationship between the distribution of the rural population and the distances to the urban areas. The q-value of the natural growth rate in 2010 was only 0.03, which indicated that the impact of the natural growth rate on the spatial pattern of rural population was minimal.

### The results of the interaction detectors

The factor detectors analyze to what degree the spatial stratified heterogeneity of the rural population distribution can be explained by an individual factor X1 by calculating the q value of X1. The interaction detectors are used to evaluate whether two factors X1 and X2 work independently or not. That is, it was evaluated whether that the factors X1 and X2 work together will increase or decrease the explanatory power to the rural population distribution patterns, comparing to only one factor X1 or X2. Using the interaction geographical detector the results are shown in Table 6. By comparing the q-value of the individual factor with the q-value after the interaction, it was found that with the interaction of any two variables, the explanatory power on the spatial distribution patterns of the rural population was greater than the independent effect of each variable. With the interaction between the natural background factors and the location factors, the explanatory power of the variables on the spatial pattern of the rural population was greatly improved. This indicated that the natural and location factors were very important factors that affected the formation of the rural spatial patterns.

**Table 6:** The q-value of interaction factors affecting rural population distribution in China.

	X1	X2	X3	X4	X5	X6	X7	X8	X9	X10	X11	X12	X13
X1	0.45												
X2	0.49	0.32											
X3	0.51	0.40	0.37										
X4	0.63	0.67	0.65	0.22									
X5	0.49	0.35	0.40	0.31	0.05								
X6	0.49	0.38	0.45	0.43	0.28	0.23							
X7	0.55	0.43	0.46	0.45	0.33	0.35	0.25						
X8	0.49	0.35	0.40	0.27	0.12	0.29	0.40	0.04					
X9	0.51	0.38	0.43	0.36	0.21	0.30	0.31	0.19	0.12				
X10	0.48	0.44	0.44	0.35	0.19	0.33	0.34	0.21	0.26	0.17			
X11	0.55	0.46	0.49	0.41	0.30	0.38	0.41	0.27	0.30	0.33	0.22		
X12	0.49	0.38	0.44	0.27	0.12	0.28	0.33	0.10	0.19	0.20	0.26	0.03	
X13	0.54	0.48	0.51	0.45	0.39	0.38	0.43	0.40	0.42	0.40	0.45	0.37	0.34

With the interaction between the potential crop productivity and the other factors, the q-value is always approximately 0.5. That is, the interaction between the potential crop productivity and the other factors could explain approximately 50% of the spatial distribution pattern of the rural population. The q-value could reach 0.63, especially with the interaction between the potential crop productivity and the cropland acreage per capita. With the interaction between the average slope and the distance to the nearest prefecture-level city, the q-value was 0.44. With the interaction between the average slope and the distance to the nearest provincial capital, the q value was 0.46; with the interaction between the degree of surface fragmentation and the distance to the nearest prefecture-level city, the q value was 0.44; and with the interaction between the degree of surface fragmentation and the distance to the nearest provincial capital, the q-value was 0.49. With the interaction between the farmland production potential and the distance to the nearest prefecture-level city, the q value was 0.48; with the interaction between the farmland production potential and the distance to the nearest provincial capital, the q-value was 0.55. With the interaction between the cultivated land area and the distance to the nearest prefecture-level city, the q value was 0.35; with the interaction between the cultivated land area and the distance to the nearest provincial capital, the q value was 0.41. The two factors of superior natural background and close distance to the city can explain the distribution patterns of the rural population in approximately 40% to 50% of cases; these are the two significant factors affecting the distribution of the rural population.

## Conclusions

Based on the geographical detector method, this paper analyzed the evolution and influencing factors of the spatial distribution pattern of China's rural population. The main conclusions are as follows:

(1) In terms of the spatial distribution, the characteristics of the spatial agglomeration of rural population were significant. The rural population were mainly distributed in plains, basins and deltas with more favorable topographical conditions. However, the degree of agglomeration of the rural population was below that of the total population. The rural population in China tended to obviously concentrate around the cities. The dense rural population areas were basically consistent with the locations of the current major urban agglomerations in China. Approximately 74% and 91% of the rural population were concentrated in the areas with 60-minute and 90-minute distances to the prefecture-level cities, respectively. The rural population centers presented circle agglomerations that decreased in density from the inside to the outside, revealing that rural development can rely on cities and that special attention to these areas is required for the implementation of China’s rural revitalization strategy.

(2) In terms of spatiotemporal evolution, the long-term large-scale rural-urban migration had not substantially changed the spatial distribution pattern of the rural population in China. From 1990 to 2010, the North China Plain, the middle and lower reaches of the Yangtze River and the Sichuan Basin had been the agglomeration centers of the rural population. However, the concentrations of the rural population were decreasing. From 1990 to 2010, the proportion of the rural population in the top 30% of counties has declined at differing degrees. The density of the rural population shifted from medium-high levels to medium-low levels.

(3) In terms of the influencing factors, compared with the research on the general influencing factors of the population spatial distribution, this paper considered natural factors and location factors from the perspective of the particularities of rural areas. The natural factors, such as the potential crop productivity, surface fragmentation degree and average slope, were the most important factors affecting the spatial distributions of the rural population. Under the combined effect of the natural factors and the distance of the location to a city, more than 40% of the spatial distribution of the rural population was explained. The current distribution pattern of the rural population was significantly influenced by the original urbanization pattern. The population migration and natural growth under the background of rapid urbanization could reshape the spatial distribution pattern of rural population, but the pattern has not yet been fundamentally changed.

The research results showed that the spatial patterns of rural population have been mainly affected by the nature environmental factors and the original spatial pattern, and the cities are important references for the spatial distribution of the rural population. Therefore, during the process of implementing China’s rural revitalization strategy and solving rural problems, the role of the nature environmental factors and the spatial distribution patterns of the original rural population must be addressed in rural governance. On the other hand, the relationship between the distribution of the rural population and cities suggests that compelling integrated development in urban-rural regions is an effective measure for solving rural problems.
